# Genome Sequence of a Subgenotype 1a Bovine Viral Diarrhea Virus in China

**DOI:** 10.1128/genomeA.01280-16

**Published:** 2016-11-10

**Authors:** Shandian Gao, Junzheng Du, Zhancheng Tian, Shanshan Xing, Jianxun Luo, Guangyuan Liu, Huiyun Chang, Hong Yin

**Affiliations:** a State Key Laboratory of Veterinary Etiological Biology, Lanzhou Veterinary Research Institute, Chinese Academy of Agricultural Sciences, Xujiaping, Lanzhou, Gansu, People’s Republic of China; b Jiangsu Co-Innovation Center for Prevention and Control of Important Animal Infectious Diseases and Zoonoses, Yangzhou, People’s Republic of China

## Abstract

A bovine viral diarrhea virus (BVDV), GS5, of the BVDV-1a subgenotype was isolated from dairy cattle in Gansu Province, northwest China. Its near-full-length genome was determined to be closely related to an early Belgian BVDV-1a strain, WAX-N, but the relatedness to domestic strains is relatively low, indicating that different genetic evolution occurred between the viral strains in cattle in China.

## GENOME ANNOUNCEMENT

Viruses of the genus *Pestivirus* within the family *Flaviviridae* comprise four recognized species, namely, bovine viral diarrhea virus type 1 (BVDV-1), BVDV-2, border disease virus (BDV), and classical swine fever virus (CSFV). Although under natural conditions, BVDV infects mainly cattle, it has been also isolated from animals other than cattle, including sheep, goats, swine, yaks, deer, and members of the Camelidae family (1–3). BVDV-1 is the dominant genotype worldwide, and at least 21 subgenotypes (BVDV-1a to BVDV-1u) have been proposed (4, 5). At least 11 subgenotypes of BVDV (BVDV-1a to -1d, BVDV-1m to -1q, BVDV-1u, BVDV-2a, and BVDV-2b) are circulating among multiple domestic animals in China (5–10). However, the complete genome of domestic isolates was scarcely reported for the classical subgenotypes except for BVDV-1b and BVDV-1d (11–13). In this study, the genome of a Chinese isolate, GS5, which belongs to the BVDV-1a subgenotype, was determined for the first time.

BVDV GS5 in passage cultures was used for viral RNA extraction and reverse transcription-PCR (RT-PCR), using primers that were designed based on the sequences of BVDV strain NADL (GenBank accession number AJ133739). The PCR products were puriﬁed and sequenced (Sangon Biotech, Shanghai, China). The near-full-length genome of GS5 is 12,189 nucleotides (nt) in length. Flanked by a 277-nt 5′ untranslated region (UTR) and a 215-nt 3′ UTR (partially determined), the open reading frame (ORF) is 11,697 nt in length and encodes 3,898 amino acids. Neither a cellular sequence insertion nor duplication of viral protein-coding regions was observed in the viral genome. Blast analysis with the genome sequence revealed that the GS5 strain shared high nucleotide identity (88 to 92%) with the BVDV-1a strains available in GenBank, followed by other subgenotypes of BVDV-1 (80 to 84%). However, it had lower nucleotide identity with strains of BVDV-2 (73%) and atypical bovine pestiviruses (71%). An individual coding region was demonstrated to have the highest nucleotide identity with an early Belgian BVDV-1a strain, WAX-N, ranging from 88.6% to 96.0%, followed by the North America strain C24V, with 88.6 to 93.9% nucleotide identity. The conserved 5′ UTR has 91.1 to 96.0% identity with reference BVDV-1a strains, but the similarity is lower (69.7 to 88.6%) than other subgenotypes of BVDV-1, BVDV-2, and atypical pestiviruses. The coding nucleotides of the immunodominant envelope protein E2 were analyzed, and GS5 is divergent from early North America strains C24V, SD1, and NADL, demonstrating 84.5 to 92.5% nucleotide identity.

Compared with domestic BVDV-1a isolates, GS5 was demonstrated to have nucleotide identity with SH1060 (93.6%) (GenBank accession no. JN248741) and HN01 (90.8%) (GenBank accession no. JX878887) in the 5′ UTR, with two camel origin BVDV isolates (88.1% and 87.9%) (GenBank accession no. KC207068 and KC207069) in the N^pro^ coding region, and with GS24 (87.1%) in the E2 coding region (GenBank accession no. KF048838). The genetic relatedness of GS5 to domestic BVDV strains that were identified recently is comparatively lower than that with the Belgian strain WAX-N or early North American strains, suggesting that GS5 may share recent common ancestor with early North American strains, indicating different genetic evolution among different BVDV strains in cattle in China.

### Accession number(s).

The genome sequence of BVDV GS5 has been deposited in GenBank under the accession number KJ541471.
